# PVDF/Polypyrrole Composite Ultrafiltration Membrane with Enhanced Hydrophilicity, Permeability, and Antifouling Properties for Efficient Crude Oil Wastewater Separation

**DOI:** 10.3390/polym17192566

**Published:** 2025-09-23

**Authors:** Banan Hudaib, Rund Abu-Zurayk, Asma Eskhan, Muayad Esaifan

**Affiliations:** 1 Chemical Engineering Department, Faculty of Engineering Technology, Al-Balqa Applied University, Amman 11134, Jordan; 2 Hamdi Mango Center for Scientific Research, The University of Jordan, Amman 11942, Jordan; 3 Nanotechnology Center, The University of Jordan, Amman 11942, Jordan; 4 Department of Chemistry, Faculty of Arts and Sciences, University of Petra, Amman 11196, Jordan

**Keywords:** PVDF, ultrafiltration, polypyrrole, composite membrane, oily wastewater, antifouling, hydrophilicity

## Abstract

The treatment of oily wastewater poses a significant environmental challenge, creating a demand for advanced separation technologies. Membrane technologies, especially ultrafiltration (UF), offer a promising solution. A novel composite polyvinylidene fluoride (PVDF) and polypyrrole (PPy) membrane was created via an in situ polymerization method, which enhances the membrane’s functionality by combining the chemical stability of PVDF with the outstanding properties of PPy, through a simple two-step process that decreases manufacturing costs. The PPy content in the PVDF matrix varies from 0 to 1.5 wt%. The membranes were analyzed for their structure, morphology, hydrophilicity, porosity, mechanical strength, flux, oil rejection, and antifouling performance. Fourier-transform infrared spectroscopy (FTIR) confirmed the successful integration of PPy, which increased hydrophilicity; the contact angle dropped from 68° for pure PVDF to 55.6° at a 1.5% PPy concentration. Scanning electron microscopy (SEM) images showed an evident increase in surface porosity and macrovoid formation; calculated porosity increased from 59.5% to 79.9%, and the hydraulic pore size increased from 2.8 nm to 28.5 nm with 1.5% PPy. Although porosity improved, mechanical strength decreased due to the formation of voids. The enhancement in hydrophilicity and porosity resulted in improved flux recovery (FR), with the PP-1 membrane achieving 93% FR and 93% fouling resistance (Rt), indicating an optimal balance for practical use. These modified membranes successfully reduce fouling, making them easier to clean in oil–water separation applications. PP-1 showed only a reduction in flux but maintained an oil rejection rate over 99%, demonstrating high stability. This combination of PVDF’s durability and PPy’s functionality makes a cost-effective, high-performance membrane that transforms oil/water separation processes for sustainable water security.

## 1. Introduction

The treatment of oily wastewater (OWW) has become a significant environmental priority worldwide, particularly in water-scarce regions such as Jordan, where freshwater resources are scarce [1]. With an annual water availability of less than 100 m^3^ per capita—far below the 500 m^3^ threshold defining absolute water scarcity—Jordan faces acute challenges in managing industrial effluents while preserving its dwindling water supplies [1]. The discharge of untreated OWW from petrochemical, food processing, and metallurgical industries exacerbates this crisis, contaminating vital groundwater aquifers and surface water bodies that provide over 60% of the nation’s potable water [2,3].

Globally, OWW comprises a complex mixture of free-floating oils (>150 µm), emulsified droplets (<20 µm), and dissolved organic compounds, including phenols and hydrocarbons [2]. Conventional treatment methods like gravity separation and chemical coagulation fail to remove emulsified oils, which represent 40–60% of industrial OWW and persistently threaten aquatic ecosystems [2]. In Jordan, where 80% of industrial facilities are concentrated in water-stressed northern regions, these limitations increase environmental risks [2,3].

Membrane technologies, particularly ultrafiltration (UF), have emerged as an applicable solution, offering rejection rates of over 95% for oil [4]. However, membrane fouling remains a significant obstacle, reducing efficiency by 30–50% in Jordan’s high-salinity OWW (TDS > 5000 ppm) [5], basically due to inorganic scaling and organic adhesion. Recent advances in nanocomposite membranes, such as PVDF modified with carbon nanotubes or zwitterionic polymers, have shown promising results for Jordan’s context, demonstrating 80% lower fouling rates in pilot studies [5,6].

The treatment of oily wastewater has emerged as a significant environmental concern due to the increasing spread of industrial activities and strict discharge regulations [6,7]. Membrane technology, particularly ultrafiltration (UF), has obtained prominence as an effective solution for oil–water separation, offering superior performance compared to conventional methods such as dissolved air flotation and chemical precipitation [7]. Recent advancements in membrane materials and modification techniques have significantly improved separation efficiency while addressing the continuous challenge of membrane fouling [8].

Polymeric membranes based on PVDF have become one of the materials of choice for treating oily wastewater due to their excellent chemical resistance and mechanical stability [5]. However, their high hydrophobicity (water contact angle > 110°) leads to rapid fouling, with studies reporting flux declines exceeding 60% within 8 h of operation [9]. This limitation has driven wide research on modification strategies, with nanocomposite approaches showing promise. Hudaib et al. [4] developed a novel PVDF membrane incorporating multi-walled carbon nanotubes (MWCNTs) and (PPy), which accomplished 98.4% oil rejection with only 12% flux decline over 50 h of continuous operation. The success of this approach stems from the excellent dispersion of pyrrole and MWCNT in the MWCNT/PPy complex, which is reflected in enhanced hydrophilicity, porosity, and permeability [4].

Surface functionalization techniques have also demonstrated remarkable success in improving membrane performance. Abdullah et al. [5] showed that single-step hydrophilization can reduce the contact angle of PVDF membranes to 28°, thereby extending the operational lifespan by 300% during treatment of palm oil mill effluent. Further advancements by Kiamehr et al. [10] through diamond-like carbon (DLC) nanostructure coatings achieved simultaneous improvements in hydrophilicity (contact angle of 15°) and mechanical strength (a 40% increase in tensile strength). These modifications address the critical need for durable membranes that maintain performance under industrial operating conditions.

Fouling mitigation remains a central focus of membrane research, with three primary strategies showing particular effectiveness [7,9]. Electrochemical repulsion using conductive membranes incorporating polypyrrole (PPy) demonstrates 80–85% lower irreversible fouling when applying a 1.5 V potential [11].

Despite these advancements, significant challenges persist in membrane technology for treating oily wastewater. The scalability of nanomaterial-enhanced membranes is limited by complex fabrication processes (5–7 steps) that increase production costs by 35–60% compared to conventional membranes [12]. Chemical resistance is another critical issue, as frequent cleaning with alkaline and acidic solutions (typically 0.1M NaOH/0.2M HCl) degrades functional coatings, reducing membrane lifespan by 40–50% [13]. These challenges underscore the need for innovative solutions that strike a balance between performance, durability, and cost-effectiveness.

The current study addresses these limitations by fabricating a novel PVDF-PPy composite membrane through in situ polymerization. This approach combines the chemical stability of PVDF with the functionality of PPy through a simplified two-step fabrication process, which is less complex and expected to be more cost-effective than multi-step nanomaterial methods [12,14]. The integrated literature demonstrates that while membrane technology has made significant strides in treating oily wastewater, opportunities remain for developing cost-effective, durable solutions that maintain enhanced separation efficiency under industrial operating conditions.

## 2. Experimental Work

### 2.1. Material

PVDF (average Mw ~534,000, powder, white) was supplied by Merck, (Burlington, MA, USA), and Ammonium peroxydisulfate (APS) (≥98%, crystalline) and the pyrrole monomer (98%, purified by distillation) were obtained from Sigma-Aldrich (St. Louis, MO, USA). N, N-Dimethylformamide (DMA) (99.8%, anhydrous) was purchased from Merck (Burlington, MA, USA), and crude oil utilized in this study was obtained from the Jordan Petroleum Refinery (Amman, Jordan).

### 2.2. Membrane Preparation

The PVDF/PPy ultrafiltration (UF) membranes were fabricated by the phase inversion method, with different pyrrole concentrations (0.25, 0.5, 1.0, and 1.5 wt%). The PVDF was dissolved in DMA at 60 °C until a homogeneous mixture was created; then, the pyrrole and APS solutions were added to the mixture. The mixture was then agitated for 24 h at 60 °C to make a casting solution, which was degassed for 2 h to expel air bubbles. Finally, the resultant blend was cast onto a glass plate by a knife blade at a thickness of 250 µm. The film was then immediately immersed in a coagulation bath of distilled water at room temperature for phase inversion to take place. The formed membrane was left in the water bath for 24 h to ensure complete solvent exchange, after which it was used.

**Figure 1:**
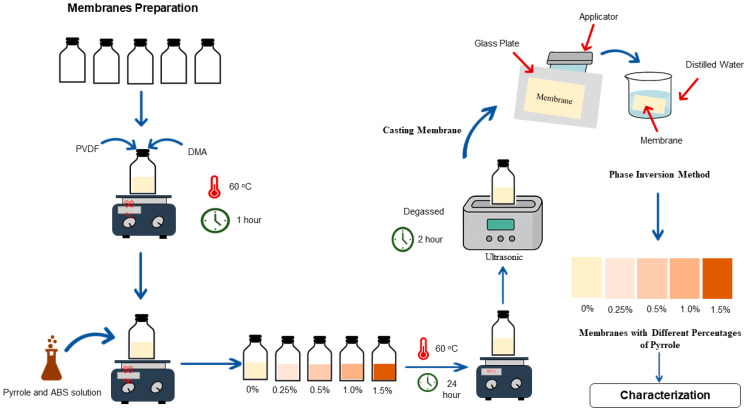
Schematic illustration of the membrane fabrication procedure by phase inversion.

Table 1 presents the details of the prepared membranes, and the procedure for membrane preparation is shown in Figure 1.

### 2.3. Oil Preparation

To prepare a crude oil solution, 0.75 mL of crude oil was mixed with 0.75 mL of polyoxyethylene-80 surfactant in 1 L of distilled water. The resulting combination was then stirred forcefully for 48 h to attain a homogeneous distribution of the crude oil. The emulsion was then examined using a UV-Vis spectrophotometer (Varian Cary 100 Bio; Agilent Technologies, Santa Clara, CA, USA), which showed a major absorbance wavelength of 358 nm. The droplet size characterization was also performed using a Zetasizer Ultra (Tempus; Malvern, UK), whose analysis revealed a peak intensity of 96.1% at a droplet size of 400.1 nm and a polydispersity index (PDI) of 0.21. This showed a stable and relatively monodisperse emulsion suitable for filtration testing.

### 2.4. Membrane Characterization and Analysis

The resultant fabricated membranes were characterized, and the comprehensive set of analytical techniques outlined below was employed.

#### 2.4.1. Material Verification (FTIR)

The incorporation of polypyrrole (PPy) into the modified membranes was assessed through Fourier-transform infrared spectroscopy (FTIR) examination, using a PerkinElmer Spectrum Two™ (Waltham, MA, USA) model to study characteristic chemical bonds. Spectra were recorded in the range of 4000–400 cm^−1^ with a resolution of 4 cm^−1^ and 32 scans per sample. The results were processed using the Spectrum™ software suite (PerkinElmer; version 10.6.2).

#### 2.4.2. Surface Hydrophilicity

Membrane surface hydrophilicity was characterized by measuring the contact angle with deionized water using a Theta Lite goniometer (Biolin Scientific; Gothenburg, Sweden). Sessile droplets of 2.5–5 µL were used for membrane samples, and three independent contact angle readings were measured. The average contact angle value was then calculated to represent the membrane’s surface wettability.

#### 2.4.3. Membrane Porosity and Pore Size

The porosity of the prepared membranes was measured via the gravimetric method. The fabricated samples were initially saturated with distilled water and then dried in a vacuum oven at 70 °C for 1 h. The porosity was subsequently calculated based on the removal of water from the wet membrane after drying, using Equation (1) as follows:

(1) $$\varepsilon =\frac{\left(W_{W}-W_{d}\right)/\rho_{water}}{\left(W_{W}-W_{d}\right)/\rho_{water}+W_{d}/\rho_{p}}$$

where

W_w_ is the wet membrane in grams (g).

W_d_ is the dry membrane in grams (g).

ρ_water_ is the density of pure water, taken to be 0.998 (g·cm^−3^).

ρ_p_ is the density of the polymer. Given the minimal inorganic content within the membrane matrix, ρ polymer was approximated to 1.78 g·cm^−3^.

The average pore size (r), which is the hydraulic pore size of the selective skin layer, can be estimated using the following relationship (Equation (2)) [15]:

r = [8 × (2.9 − 1.75ε) × η × L × F/(3600 × ε × ΔP)]^1/2)^ (2)

Here,

r is expressed in meters (m).

η is the dynamic viscosity of water, equal to 8.9 × 10^−4^ Pa·s.

L is the membrane thickness (m).

F is the pure water flux (m^3^/m^2^·h).

ε is the membrane porosity (dimensionless).

ΔP is the applied transmembrane pressure (Pa).

The average pore radius (r) estimated from Equation (2) represents the adequate hydraulic pore size of the selective skin layer.

In addition to this theoretical estimation, the average apparent pore size was also statistically measured from SEM images, where image analysis was performed on selected membrane surface areas to validate the calculated values.

#### 2.4.4. Morphological and Mechanical Characterization of Modified Membranes

The membrane samples’ surface and cross-sectional structure were examined by a field-emission scanning electron microscope (FE-SEM, Quanta FEG 450, FEI, Hillsboro, OR, USA). The membrane specimens were carefully fractured in liquid nitrogen and then sputter-coated with conductive gold before imaging. Subsequently, the mechanical integrity and tensile strength of the membranes were studied using an electromechanical universal tensile testing machine (BMT-E Series, TESTometric Co., Ltd., Rochdale, UK).

#### 2.4.5. Permeation and Antifouling Performance

The permeability and oil rejection capabilities of the membranes were evaluated using a high-pressure stirred cell (HP 4750, Sterlitech, Auburn, Washington, DC, USA) with an effective membrane area of 14.6 cm^2^. A constant stirring speed of 300 rpm was maintained to minimize concentration polarization. All experiments were conducted at room temperature (23 ± 2 °C) and at a constant feed pressure of 1 bar. The concentrations of crude oil for the permeation and feed solutions were determined using a UV spectrophotometer (Varian Cary 100 Bio; Agilent Technologies, Santa Clara, CA, USA) at a central wavelength of 358 nm.

Both flux and oil rejection were calculated using the following formulas (Equations (3) and (4), respectively):

(3) $$Flux=\frac{V_{m}}{A_{m}\times t}$$

where

$V_{m}=$ permeate volume.

$A_{m}=$ membrane surface area.

t = time interval.

(4) $$Rejection=\left(1-\frac{C_{p}}{C_{0}}\right)\times 100\%$$

where

*C_P_* and *C*_0_ are concentrations of oil for the permeation and feed solution (mg/L).

Membrane fouling refers to the accumulation of substances on or within a membrane’s pores. In this study, crude oil was employed as a foulant model to evaluate the antifouling performance of the modified membranes. The testing procedure involved three main steps: first, the membrane’s initial water permeability was assessed using pure water; next, it was exposed to crude oil to simulate fouling conditions; finally, the membrane was subjected to a cleaning protocol—washing with 0.1 M sodium hydroxide solution for half an hour, followed by rinsing in pure water for 15 min. After cleaning, the pure water flux was measured again. The flux recovery ratio (FRw) and total fouling ratio (Rt) were then determined using the corresponding standard equations, Equations (5) and (6) [4]:

(5) $$FRw=\left(\frac{J_{c}}{J_{0}}\right)\times 100$$

(6) $$R_{t}=\left(1-\frac{J_{oil}}{J_{0}}\right)\times 100$$

where $J_{0\;}$ is water flux, $J_{c}$ is the water flux of the cleaned membrane, and $J_{oil}$ is the crude oil solution flux.

## 3. Results and Discussion

### 3.1. FTIR Analysis and Implications for Oil Removal

**Figure 2:**
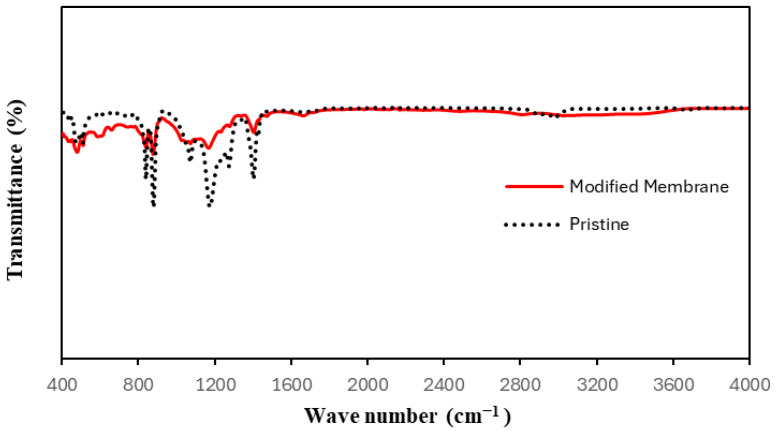
FTIR spectra of the modified and pristine membranes.

Fourier-transform infrared spectroscopy confirmed the successful modification of the PVDF membrane with polypyrrole (PPy), as shown in Figure 2.

The FTIR spectra of the pristine PVDF membrane exhibit characteristic absorption bands at ~1171 cm^−1^ (–CF_2_ symmetric stretching) and ~1396 cm^−1^ (–CH_2_ bending), and a lower-frequency β-phase peak at approximately 880 cm^−1^ (C–C–C asymmetrical stretching), consistent with typical crystalline PVDF signatures [16,17]. In terms of pyrrole functionalization (PPy/PVDF composite), new spectral features exist: a broad N–H stretching band around 3450 cm^−1^ appears, indicative of polypyrrole’s amine functionality, and distinct peaks at ~1631 cm^−1^ and ~1544 cm^−1^ correspond to the characteristic C=C and C–C ring-stretching modes of pyrrole polymer chains [18]. Importantly, both pristine and modified membranes retain the PVDF backbone bands, confirming composite formation without polymer degradation [18].

The presence of these PPy-related absorption bands, which are absent in the pristine spectrum, provides evidence of successful incorporation of polypyrrole onto the PVDF matrix. Moreover, the minor shifts in the PVDF CF_2_-related peaks suggest interactions—possibly hydrogen bonding or π–π coupling—between PPy and PVDF domains, leading to interfacial compatibility without altering PVDF’s structure. These FTIR results suggest effective functionalization of PVDF with pyrrole-derived moieties.

### 3.2. Integrated Analysis of Hydrophilicity, Porosity, Pore Size Morphology, and Flux Enhancement in PPy-Modified PVDF Membranes

**Figure 3:**
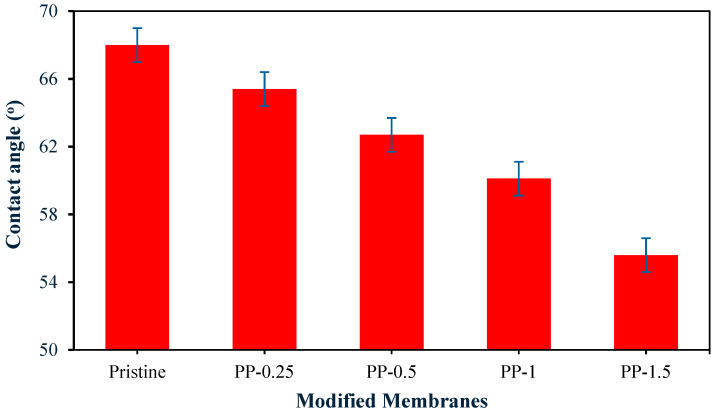
Modified membrane hydrophilicity.

The observed reduction in water contact angle (from 68° for pure PVDF to 55.6° at 1.5% PPy), as can be seen in Figure 3, confirms significantly enhanced membrane hydrophilicity after polypyrrole (PPy) incorporation.

This phenomenon is attributed to PPy’s polar functional groups (–NH–), which introduce hydrogen-bonding sites that improve surface hydration and wettability. On the other hand, (PPy) addition into (PVDF) matrices also enhances membrane porosity and pore size through synergistic mechanisms such as PPy hydrophilic functionalities, which enhance increased solvent–nonsolvent exchange during phase inversion, support improved rapid precipitation of polymers and macrovoid formation, and thereby support overall porosity and pore size [19]. As shown in Table 2, the porosity increased from 59.5% for the pristine membrane to a maximum of 79.9% for PP-1, and pore size increased from 2.8 nm for the pristine membrane to a maximum of 28.5 nm for PP-1.5. Moreover, PPy also serves as a pore-former: its aggregates make nucleation sites, while leachable oligomers create micropores in solvent evaporation [20].

Concurrently, water flux measurements are expected to show an increase in permeability with increasing PPy loading.

**Figure 4:**
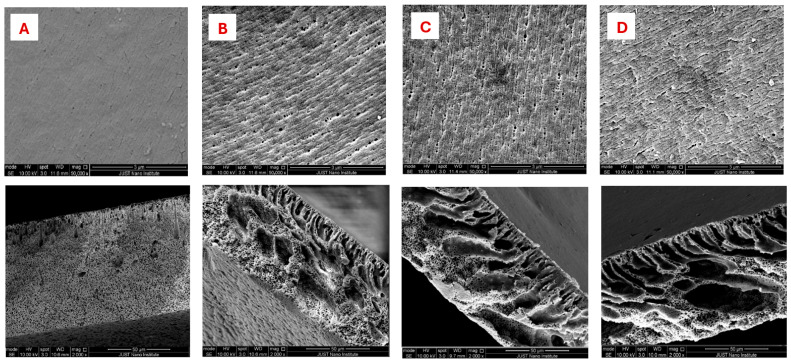
SEM images of top and cross-sections of pristine and modified membranes; (**A**) pristine PVDF, (**B**) PP-0.5, (**C**) PP-1, and (**D**) PP-1.5.

The surface morphologies of the selected membranes were characterized using scanning electron microscopy (SEM). A comprehensive analysis of the morphological changes was conducted by examining both the surface and cross-sectional morphologies. Figure 4 displays the surface and cross-sectional images of the fabricated membranes with varying polypyrrole concentrations. The SEM images in Figure 4 show fundamental changes in the membranes’ morphology after polypyrrole (PPy) addition.

Pristine membranes exhibit a characteristic asymmetric structure with a thick, dense skin layer and limited sub-layer porosity, resulting in sponge-like regions that impede water permeation (Figure 4A). Upon adding 0.5% PPy, accelerated nonsolvent-induced phase separation (NIPS) occurs due to PPy’s hydrophilic nature, which causes instability in the casting solution. This enhances rapid solvent–nonsolvent (water) exchange, yielding thinner skin layers and elongated finger-like macrovoids, and cross-sectional SEM further verifies these structural transitions, showing enhanced pore interconnectivity across the membrane’s depth [19].

**Figure 5:**
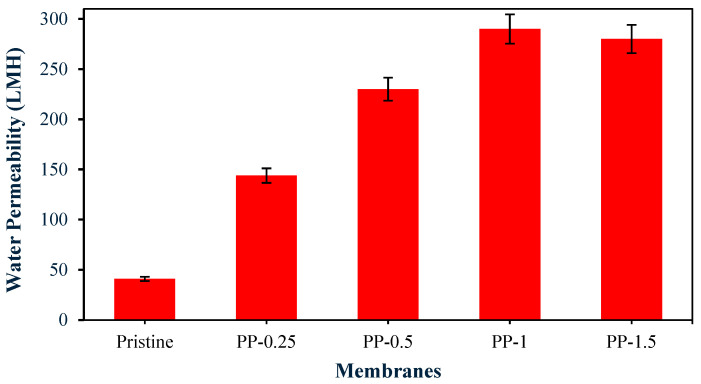
Water permeability for pristine and modified membranes.

As shown in Figure 5, the membrane’s flux improved with the integration of polypyrrole (PPy). The pristine membrane had a very low water flux of 40.9 LMH (liters per square meter per hour), but this increased to approximately 130 LMH when 0.25% PPy was added. The highest flux was observed with the PP-1-modified membrane, which achieved a flux of 290 LMH. Thus, this increase in membrane flux is mechanistically explained by three interlinked factors: There is increased hydrophilicity and hydration from PPy’s polar groups, which form hydration layers that lower transmembrane energy barriers and reduce the hydrophobic adsorption of foulants. Furthermore, morphological optimization, characterized by thinner skin layers and enlarged pore channels (with increased pore diameter), reduces hydraulic resistance [21]. Macrovoid elongation creates continuous pathways for water permeation, while higher surface porosity increases filtration area.

Additionally, the accelerated phase separation of PPy amplifies solvent–nonsolvent diffusion kinetics during NIPS, thereby suppressing delayed demixing that causes denser morphologies. This aligns with Jalali et al.’s findings [20], which show that hydrophilic additives reduce skin layer thickness by more than 35% in polysulfone systems.

A comparison of results of this study with similar studies in the literature that used PVDF membranes for removing oil from wastewater is shown below in Table 3.

The PVDF/PPy membrane offers a remarkable combination of good water permeability at 290 LMH and excellent oil rejection of over 99%.

### 3.3. Tensile Performance of Polypyrrole-Modified PVDF Membranes

**Figure 6:**
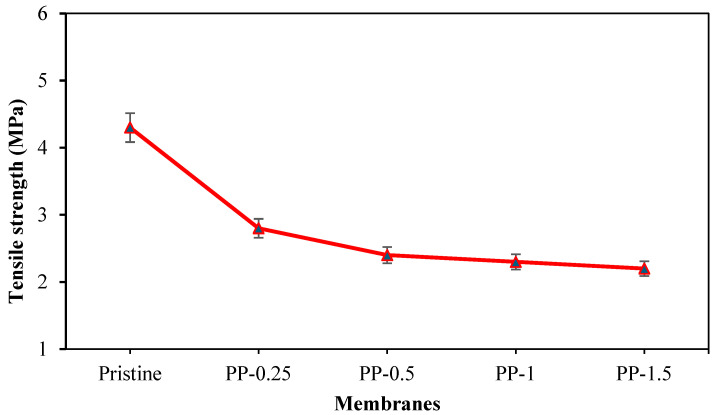
Tensile strength for pristine and modified membranes.

During high-pressure filtration operations, the tensile strength of PVDF membranes is a critical mechanical parameter that maintains their structural integrity. The tensile strength for the fabricated membranes is illustrated in Figure 6.

It is expected that the incorporation of pyrrole will decrease the tensile strength of the PVDF matrix, as a result of increased porosity and pore size, which means increased space between polymer chains, allowing for more effortless molecular movement and further contributing to tensile strength decrease. Moreover, the stress is not evenly distributed across the entire cross-section when a material is stretched. The presence of pores creates stress concentrations around the void edges, making the material more prone to failure at lower overall stress levels. Similar results were found by Kloster et al. (2023), who found that the tensile modulus was highly inversely correlated with the porosity of PVDF [27].

Another contributory factor to the reduction in the tensile strength of PVDF is the fact that the tensile strength of pyrrole is lower than that of PVDF.

### 3.4. Membrane Total Fouling, Stability, and Cleaning

Membrane performance in membrane separation and water treatment faces a significant obstacle: membrane fouling. This phenomenon involves the buildup of undesirable substances, such as oil droplets and emerging contaminants, either on the membrane’s surface or within its pores [28]. The consequences include increased energy usage, reduced water flow (flux), a shorter membrane lifespan, and higher operating expenses [29].

**Figure 7:**
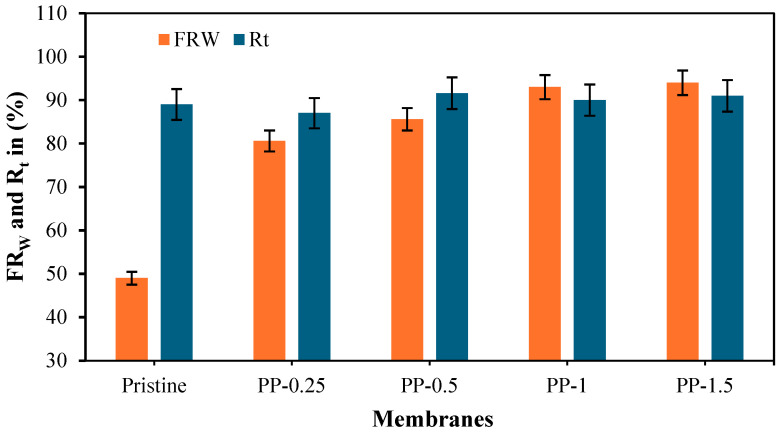
Fabricated membranes: flux recovery and total fouling.

To analyze the improvements in flux recovery from oil for caustic soda cleaning agents using modified membranes, experiments were conducted on both pristine and fabricated membranes. Results for flux recovery and the total fouling ratio are presented in Figure 7.

The flux recovery (FWR) and total fouling ratio (Rt) of the fabricated membranes revealed a critical trade-off between cleanability and fouling resistance, as shown in Figure 7 [4]. While the pristine membrane exhibited poor flux recovery (FWR = 49%) and significant fouling (Rt = 89%), surface modification with (PPy) enhanced cleanability, with FWR increasing progressively from 80.6% (PP-0.25) to 94% (PP-1.5). However, higher PPy loadings increased fouling resistance: PP-0.5, PP-1, and PP-1.5 increased Rt to 91.6%, 90%, and 91%, respectively. Notably, PP-1 achieved near-optimal FWR (93%) with acceptable fouling (Rt = 90%), representing the best compromise for practical applications. These results suggest that modified membranes optimize the balance between reversible fouling mitigation and cleaning efficiency in oil/water separation.

**Figure 8:**
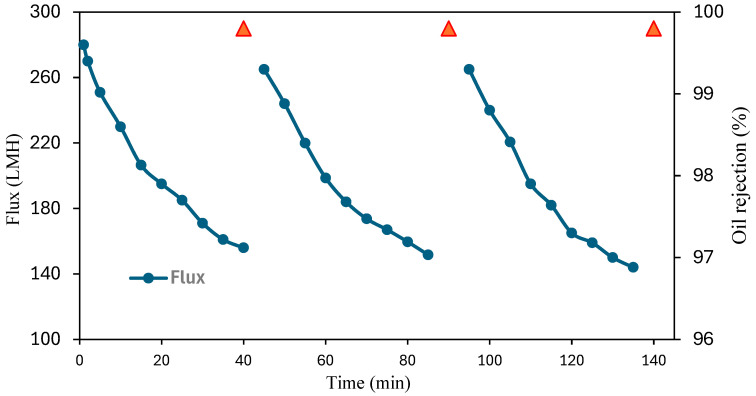
Membrane flux and oil rejection recycling for PP-1 versus time in three filtration cycles after cleaning.

To study membrane stability, the PP-1 membrane underwent filtration and rejection tests for approximately 140 min, as shown in Figure 8. The membrane’s oil rejection efficiency was measured every 40 min, followed by a caustic soda wash and subsequent reuse.

The results revealed a minor decline in flux for the modified membrane, while maintaining an oil rejection efficiency of more than 99%. This suggests that the modified membranes possess high stability.

## 4. Conclusions

This study presents a cost-effective and straightforward method for enhancing oil/water separation performance by successfully fabricating PVDF/PPy composite ultrafiltration membranes using an in situ polymerization method. The integration of PPy into the PVDF matrix significantly increased membrane hydrophilicity, as shown by a decrease in contact angle from 68° to 55.6° at 1.5 wt% PPy. The enhancement in hydrophilicity and calculated porosity, which increased from 59.5% to 79.9% with pore sizes increasing from 2.8 nm to 27.3 nm, resulted in an improved flux recovery rate. Despite a slight decline in mechanical strength due to increased void formation, the PP-1 membrane achieves 93% flux recovery and 93% fouling resistance while maintaining over 99% oil rejection, reflecting an optimal balance of permeability and stability.

These results offer an efficient and easy-to-clean PVDF/PPy membrane for treating oil-contaminated water, particularly at a 1.0 wt% PPy concentration. These membranes’ outstanding features, including chemical stability, antifouling properties, and high separation efficiency, open promising industrial-scale applications in the petrochemical, food processing, and metallurgical industries, as well as wastewater treatment. Future research will focus on long-term performance and scaling up the fabrication process to meet industrial demands.

## Figures and Tables

**Table 1:** Casting solution compositions.

Membranes	PVDF (wt.%)	Pyrrole (wt.%)	APS (wt.%)	Solvent (wt.%)
**Pristine**	12	0	0	88
**PP-0.25**	12	0.25	0.25	87.5
**PP-0.5**	12	0.5	0.5	87
**PP-1**	12	1	1	86
**PP-1.5**	12	1.5	1.5	85

**Table 2:** The porosity and pore size of fabricated membranes at 250 µm thickness.

Membranes	Porosity (%)	Pore Size (nm)
**Pristine**	59.5	2.8 ± 1
**PP-0.25**	66.3	9.1 ± 1.5
**PP-0.5**	77.1	18.2 ± 2.5
**PP-1**	79.9	27.3 ± 2.7
**PP-1.5**	78.3	28.5 ± 3.1

**Table 3:** Performance comparison of modified PVDF UF membranes.

Modified Membrane	Oil Rejection (%)	Flux (LMH)	Refs.
**PVDF-SiO**	98.9	93.86	[22]
**PVDF-PVP composite**	77.5	9–185	[23]
**PVDF/SiO_2_/PVP**	94.5	198	[24]
**α-FeOOH/g-C_3_N_4_@PVDF**	99.1	452–650	[25]
**DAC/PEI-coated PVDF**	99	350	[26]
**PVDF/PPy**	<99	290	Current work

## Data Availability

The original contributions presented in the study are included in the article, further inquiries can be directed to the corresponding author.
